# Bioenergy Sorghum Crop Model Predicts VPD-Limited Transpiration Traits Enhance Biomass Yield in Water-Limited Environments

**DOI:** 10.3389/fpls.2017.00335

**Published:** 2017-03-21

**Authors:** Sandra K. Truong, Ryan F. McCormick, John E. Mullet

**Affiliations:** ^1^Interdisciplinary Program in Genetics, Texas A&M UniversityCollege Station, TX, USA; ^2^Department of Biochemistry and Biophysics, Texas A&M UniversityCollege Station, TX, USA

**Keywords:** energy sorghum, vapor pressure deficit, limited transpiration, crop model, water-limited environments, biomass, APSIM

## Abstract

Bioenergy sorghum is targeted for production in water-limited annual cropland therefore traits that improve plant water capture, water use efficiency, and resilience to water deficit are necessary to maximize productivity. A crop modeling framework, APSIM, was adapted to predict the growth and biomass yield of energy sorghum and to identify potentially useful traits for crop improvement. APSIM simulations of energy sorghum development and biomass accumulation replicated results from field experiments across multiple years, patterns of rainfall, and irrigation schemes. Modeling showed that energy sorghum's long duration of vegetative growth increased water capture and biomass yield by ~30% compared to short season crops in a water-limited production region. Additionally, APSIM was extended to enable modeling of VPD-limited transpiration traits that reduce crop water use under high vapor pressure deficits (VPDs). The response of transpiration rate to increasing VPD was modeled as a linear response until a VPD threshold was reached, at which the slope of the response decreases, representing a range of responses to VPD observed in sorghum germplasm. Simulation results indicated that the VPD-limited transpiration trait is most beneficial in hot and dry regions of production where crops are exposed to extended periods without rainfall during the season or to a terminal drought. In these environments, slower but more efficient transpiration increases biomass yield and prevents or delays the exhaustion of soil water and onset of leaf senescence. The VPD-limited transpiration responses observed in sorghum germplasm increased biomass accumulation by 20% in years with lower summer rainfall, and the ability to drastically reduce transpiration under high VPD conditions could increase biomass by 6% on average across all years. This work indicates that the productivity and resilience of bioenergy sorghum grown in water-limited environments could be further enhanced by development of genotypes with optimized VPD-limited transpiration traits and deployment of these crops in water limited growing environments. The energy sorghum model and VPD-limited transpiration trait implementation are made available to simulate performance in other target environments.

## Introduction

Predicted increases in world population and development by 2050 are projected to increase the demand for food, forage, biofuels, and bio-products from agriculture by ~50% (Fedoroff and Cohen, 1999; Bruinsma, 2009; Hall and Richards, 2013). Crop production requires substantial water resources due to the linkage between photosynthetic CO_2_ uptake/assimilation and transpirational water loss through stomata (Vadez et al., 2014). Insufficient water supply is a major cause of low crop productivity annually and periodic drought can cause large disruptions in agricultural output (Boyer, 1982; Boyer et al., 2013). Therefore, to meet future demand for agricultural products, crops with improved water capture, water use efficiency, and drought resilience are needed to enhance sustainable production (Boyer et al., 2013; Vadez et al., 2013).

*Sorghum bicolor* is a versatile drought resilient C4 grass crop that currently is used to produce grain and forage on more than ~65M ha world-wide. Sorghum crops are particularly important for subsistence farming in the semi-arid tropics (Doggett, 1988). Bioenergy sorghum is a relatively new type of sorghum hybrid crop designed for long growing seasons to enhance biomass yield (Rooney et al., 2007; Olson et al., 2012; Gill et al., 2014). The development of high biomass sorghum hybrids was initiated following discovery of a breeding system that allows production of late flowering hybrids from early flowering inbreds (Rooney and Aydin, 1999; Rooney et al., 2007). Bioenergy sorghum hybrids have high photoperiod sensitivity due to the combined action of *Ma1, Ma5*, and *Ma6* that inhibit flowering in day lengths greater than 12.4 h (Rooney and Aydin, 1999; Murphy et al., 2011, 2014; Mullet et al., 2014; Yang et al., 2014). As a consequence, energy sorghum hybrids that develop past the juvenile phase in the spring when day length are >12.4 h do not undergo floral initiation until day lengths decrease below 12.4 h in the fall. The resulting long vegetative growth duration of energy sorghum hybrids, combined with C4 photosynthesis, high radiation interception and use efficiency, and annual cropping seasons that permit rotations and adjustments for economic conditions, make energy sorghum hybrids a productive high biomass crop especially useful for water-limited growing regions (Rooney et al., 2007; Gill et al., 2014; Mullet et al., 2014).

Sorghum is amenable to genetic improvement of hybrids, due to a diverse germplasm, good genomics platform, and tractable genetics (Rooney and Aydin, 1999; Rooney et al., 2007; Mullet et al., 2014). Breeding and genetic improvement of bioenergy sorghum hybrids has been underway on a small scale for only ~15 years and as a consequence, there are many unexploited opportunities for improving the crop's productivity and resilience. One way to increase the rate of crop improvement is to use models to help identify physiological traits that have significant predicted yield benefits for optimization through genetic selection (Zhu et al., 2010; Hammer et al., 2016). This approach was used in a prior study to examine the utility of increased leaf angle in bioenergy sorghum using structure-function modeling (Truong et al., 2015). The predicted beneficial impact on biomass yield was confirmed by identifying leaf angle QTL and field-testing genotypes with varying leaf angle (Truong et al., 2015). The integration of structure-function modeling, crop-scale modeling, automated phenotyping, and quantitative genetics is expected to further accelerate crop genetic improvement.

The Agricultural Production System SIMulator (APSIM) is a crop modeling framework that enables predictions of the growth and productivity of crop species under varying environmental conditions and management practices (McCown et al., 1996; Keating et al., 2003; Holzworth et al., 2014). The sorghum module in APSIM has benefitted from continuous development and improvement since 1994 (Hammer and Muchow, 1994), incorporating established models of sorghum phenology, canopy development, growth, and nitrogen use (Sinclair et al., 1984; Rosenthal et al., 1989; Birch et al., 1990; Hammer and Muchow, 1994; Chapman et al., 2000a,b). APSIM has also been enhanced to model complex adaptive traits and genotype to phenotype predictions (Hammer et al., 2010). In addition to its record of providing accurate predictions of sorghum development (Hammer and Muchow, 1994; Kumar et al., 2009; Hammer et al., 2010; Kholová et al., 2014; Lobell et al., 2015), APSIM's modular design provides a flexible platform for examining the impact of variation in traits and physiological processes on plant development. To date, APSIM has been used predominately for modeling grain crops and additional work has demonstrated its efficacy in sugarcane (Nair et al., 2012). APSIM should be similarly effective for examining biomass accumulation in bioenergy sorghum hybrids with long vegetative growth duration. Therefore, we extended the APSIM grain sorghum model to bioenergy sorghum and examined its performance relative to experimental data from field experiments across multiple years and patterns of rainfall and water availability. Additionally, APSIM was used to investigate the potential impact of traits that limit transpiration under high vapor pressure deficit (VPD) in bioenergy sorghum.

VPD describes the difference in water vapor pressure within the leaf and the surrounding air. Since transpiration rate increases with increasing VPD, limiting transpiration under conditions of high VPD has been predicted to contribute to the water use efficiency of grain sorghum and corn (Sinclair et al., 2005; Kholová et al., 2014; Messina et al., 2015). VPD-limited transpiration traits enable water conservation and improve water use efficiency by imposing a restriction on transpiration rate when VPD becomes sufficiently large (Parent et al., 2009; Tardieu et al., 2009; McAdam and Brodribb, 2015). Previous experiments have demonstrated that VPD-limited transpiration is a genetically regulated trait in sorghum. Some genotypes display differences in the linear increase in transpiration rate with increasing VPD, whereas other genotypes display a VPD breakpoint, defined as the VPD at which the slope of the linear response between VPD and transpiration rate decreases (Gholipoor et al., 2010; Choudhary et al., 2013; Choudhary and Sinclair, 2014b; Riar et al., 2015). These sorghum genotypes respond to high VPDs by reducing their transpiration rates, effectively limiting water loss under environmental conditions that result in low transpiration efficiencies, thereby increasing overall crop water use efficiency.

The VPD-limited transpiration trait may be especially beneficial for bioenergy sorghum hybrids that are grown in regions subject to high VPD and water limitation. Any daily advantage that the trait confers would potentially be compounded over extended periods of high VPD and water deficit that occur during the crop's long growing season. Moreover, enhancing the resilience of energy sorghum by increasing the crop's tolerance of long periods of water limitation will enable the crop to utilize intermittent rainfall for growth and biomass accumulation. Furthermore, future climate change modeling predicts increases in VPD that will be detrimental to vegetation (Lobell et al., 2014; McDowell et al., 2016), therefore determining the potential beneficial impact of VPD-limited transpiration traits and potential trade-offs on energy sorghum biomass yield and resilience is of great interest.

Previous methods for incorporating a limited transpiration trait into crop models imposed a VPD-independent and a VPD-dependent maximum transpiration rate. In grain sorghum, VPD-limited transpiration was modeled as a maximum transpiration rate per unit leaf area, such that the transpiration rate would plateau at the designated maximum regardless of further increases in VPD (Sinclair et al., 2005; Kholová et al., 2014). In maize, limited transpiration was modeled as a piecewise function whereby, at or above a designated VPD (the VPD breakpoint), transpiration rate would plateau and not increase further (Messina et al., 2015). These models are characteristic of some sorghum genotypes but do not capture the full range of VPD modulated transpiration responses reported for sorghum (Gholipoor et al., 2010; Choudhary et al., 2013; Choudhary and Sinclair, 2014b; Riar et al., 2015). To capture this additional complexity, we implemented a dynamic VPD-limited transpiration modification as part of an energy sorghum model in APSIM and used this modification to examine the effects of a range of VPD-limited transpiration traits on biomass accumulation. Adapting APSIM for bioenergy sorghum and VPD-limited transpiration enables predictions of energy sorghum phenology, biomass accumulation in a range of environments and agronomic practices, and analyses of the impact of the VPD-limited transpiration on biomass accumulation.

## Methods

### Crop model simulations for energy sorghum in APSIM

The daily progression of sorghum biomass accumulation given environmental data and management practices was simulated using Agricultural Production Systems Simulator (APSIM 7.7, www.apsim.info) (McCown et al., 1996; Keating et al., 2003; Holzworth et al., 2014). Daily maximum temperature, minimum temperature, and precipitation data for College Station, TX (Latitude 30.58917, Longitude −97.36472) from the beginning of year 2000 to the end of year 2014 were obtained from the Daily Global Historical Climatology Network, GHCN-DAILY (Menne et al., 2012 access date: January 2016). The values of maximum and minimum radiation per day were obtained from “Maximum and Minimum Radiation Incident On An Equator-pointed Tilted Surface (kWh/m2/day)” from NASA Surface meteorology and Solar Energy (SSE) data set for Latitude 30.601, Longitude 96.314 (https://eosweb.larc.nasa.gov/). The tav_amp APSIM function was used to calculate annual average ambient temperature (TAV) and annual amplitude in mean monthly temperature (AMP). The soil depth parameters were adjusted to be reflective of College Station, TX, where energy sorghum root systems rarely extend beyond 1,000 mm below the soil surface. This constraint was implemented by making the 6th soil layer (1200–1500 mm depth) a water table that restricted root growth and water uptake past the 6th soil layer.

Crop management practices used for modeling were based on standard methods for growing energy sorghum (Rooney et al., 2007; Olson et al., 2012). Sowing each year was modeled to occur between April 14th and May 1st. The sowing density and plant spacing in rows reflect practices applied in College Station, TX, in 2008 and 2009 with 13.2 plants m^−2^ and 76 cm row spacing. Fertilization was simulated to apply 100 kg of nitrogen (N) per hectare based on production practices described previously (Olson et al., 2012). The unlimited irrigation regime was implemented using furrow irrigation between rows approximately every 2 weeks if no rainfall occurred and less often when rainfall provided water for plant growth (Olson et al., 2012). For simulations without VPD-limited transpiration, irrigation of 150 mm of water was applied on soil water deficit demand of 50 mm as part of three irrigation scenarios: no irrigation (other than starting with a fully saturated soil profile prior to sowing, rainfall was the only water source), limited irrigation (irrigation stops on July 7th), and unlimited irrigation (irrigation on demand during the growing season).

### Adapting APSIM to model delayed flowering due to high photoperiod sensitivity

Delayed flowering caused by very high photoperiod sensitivity is a trait that differentiates energy sorghum from most other sorghum crops. As a consequence of this trait, energy sorghum hybrids planted in central Texas in mid-April grow in the vegetative phase for ~150 days and initiate flowering in mid-September when day lengths decrease below 12.4 h (Rooney and Aydin, 1999). The implementation of photoperiod sensitivity in the APSIM sorghum module is based on extension of thermal time in the vegetative phase (Hammer et al., 2010; Holzworth et al., 2014). Therefore, thermal time (e.g., photoperiod slope) parameters were set to be consistent with observed time to floral initiation for energy sorghum hybrids such as TX08001 grown in College Station, Texas (Table 1). Modeling of energy sorghum hybrid performance other locations and latitudes could be implemented in a similar way by determining thermal time from planting until day lengths are <12.4 h.

**Table 1:** **Parameter values for modeling canopy development and growth in the energy sorghum crop model used in this study**.

**Parameter description**	**Constants-species-specific**		
	**Default**	**Adjusted**	**XML variable name**
**SECTION 1—CROP PHENOLOGY: DEVELOPMENT PARAMETERS**			
Maximum leaf number	40	50	leaf_no_max
Leaf appearance rate 1 (°Cd)	41	50	leaf_app_rate1
**SECTION 2—PHOTOSYNTHESIS BIOMASS GROWTH AND PARTITION**			
Radiation use efficiency, RUE (g MJ-1) from juvenile to floral initiation	1.25	2.3	Rue
Extinction coefficient for green leaf from row spacing 0.5 m and 1.0 m	0.40	0.70	y_extinct_coef
**SECTION 7—SENESCENCE AND DETACHMENT**			
Light delay factor for leaf senescence (days)	10.0	25.0	sen_light_time_const
Radiation level for onset of leaf senescence (MJ m-2)	2.0	0.5	sen_radn_crit
Water supply:demand ratio for onset of leaf senescence (MJ m-2)	0.25	0.03	sen_threshold
	**GENOTYPE**		
	**late maturity**	**texas_energy**	**XML variable name**
**PHENOLOGY**			
Photoperiod criteria 1	12.3	12.4	photoperiod_crit1
Photoperiod criteria 2	14.6	13.4	photoperiod_crit2
Photoperiod slope	38.6	1545	photoperiod_slope
**LEAF AREA—TPLA APPROACH**			
Curvature coefficient, α, for leaf area (1/°Cd)	0.018	0.003	tpla_prod_coef
Power coefficient, γ, for number of leaves to total plant leaf area	2.95	2.68	main_stem_coef
Inflection coefficient, β, of total plant leaf area curve	0.66	0.725	tpla_inflection_ratio
**CANOPY HEIGHT**			
Minimum weight at maximum height to calculate density of stem accumulation (g/stem)	80	230	x_stem_wt
Maximum height (m)	2.0	4.0	y_height

*The grain sorghum crop model parameters for a late maturity cultivar described in APSIM sorghum module (apsim.info) were modified to model energy sorghum. Energy sorghum phenology is approximated such that energy sorghum will initiate floral induction in mid-September given College Station, TX, environments as observed (Rooney and Aydin, 1999; Olson et al., 2012). Other parameters were modified such that the model output generally resembled measurements of TX08001 in 2008 (Olson et al., 2012)*.

### Adapting APSIM sorghum module for VPD-limited transpiration traits

In APSIM's sorghum model, daily biomass accumulation is determined primarily from two inputs: radiation energy intercepted by the plant canopy (radiation energy supply) and soil water supply. For a given day, if water supply is not limiting, the amount of radiation intercepted determines biomass accumulation. Daily plant water demand is calculated from radiation energy supply. The required amount of water for transpiration (water demand) needs to be extracted by roots from the soil profile to maximize biomass accumulation. If water available from the soil profile is less than the water required by the plant during canopy gas exchange for maximal biomass accumulation, then water supply reduces daily biomass accumulation. The amount of water utilized by the plant during gas exchange required for biomass accumulation is calculated from plant water demand when soil water supply was sufficient to meet demand, or from available water supply when supply was less than plant water demand.

To extend this model, VPD-limited transpiration was introduced into the calculation of potential change in biomass per day by impacting plant water demand as a function of VPD. Daily weather input and biomass accumulation potential were interpolated at hourly timesteps, and for each daytime hour, the daily potential for biomass accumulation restricted by water was calculated as a function of hourly VPD. This effectively introduced the ability for a plant to slow its transpiration rate under conditions of high VPD (e.g., mid-afternoon), in which case the plant would demand less water under conditions of high VPD. Consequently, this slowed the rate of biomass accumulation under conditions of high VPD and reduced the amount of water used in high VPD conditions when transpiration efficiency is low. The calculations are described below, and their implementation in the APSIM sorghum C++ module is made available (see Code Availability).

VPD-limited transpiration was modeled using the parameters {*vpd*_*BP*_, *m*_1_, *m*_2_} which are typically obtained from experiments quantifying VPD-limited transpiration (**Figure 2**, Table 2). A VPD-limited transpiration trait can be characterized by a VPD breakpoint (*vpd*_*BP*_), the slope of the linear relationship between transpiration rate and VPD for VPDs that are less than the VPD breakpoint (*m*_1_), and the slope of the linear relationship between transpiration rate and VPD for VPDs that are greater than the VPD breakpoint (*m*_2_); these three parameters can be defined in the .xml sorghum file provided by the user. Given these parameters, a daily biomass accumulation $\sum_{t=sunrise}^{sunset}B\left(t\right)$ is calculated on an hourly (*t*) basis over the course of a day (sunrise to sunset). In order to evaluate VPD-limited transpiration on an hourly basis, VPD and biomass data calculated from radiation energy supply was interpolated hourly with respect to climate data by implementing the piecewise function described by Eccel (2010) from sunrise to sunset of sinusoid I and II equations. With this calculation, for each hour, *t*, there is an hourly transpiration rate, *T*_*r*_(*t*), based on potential biomass accumulation from radiation energy, *B*_*r*_(*t*), leaf area index, *lai*, and an hourly vapor pressure deficit, *vpd*(*t*), such that the following can be evaluated (Table 2 describes variables):

**Table 2:** **Descriptions of variables in the VPD-limited transpiration model modified within the APSIM sorghum module**.

**Variable**	**Description**	**Time-scale**	**Origin**
*B*_*r*_(*t*)	Potential biomass from radiation energy interpolated from daily potential biomass calculation from radiation energy	Hour	Biomass class (APSIM)
*lai*	Leaf area index	Day	Leaf class (APSIM)
*W*_*soil*_	Soil water available from water and root class	Day	Water class (APSIM)
*TE*_*c*_	Genotype-specific, stage-dependent transpiration efficiency coefficient	Developmental stage	Parameterized (Hammer et al., 1997)
*m*_1_	The slope of transpiration rate when *vpd*(*t*) is less than *vpd*_*BP*_.	Fixed	Parameterized
*m*_2_	The slope of transpiration rate when *vpd*(*t*) is greater than or equal to *vpd*_*BP*_.	Fixed	Parameterized
*vpd*_*BP*_	Vapor pressure deficit (VPD) at which transpiration rate transitions into another linear function.	Fixed	Parameterized
*vpd*(*t*)	Vapor pressure deficit (VPD) at hour *t* is interpolated from minimum and maximum daily temperature.	Hour	VPD-limited transpiration model
*T*_*r*_(*t*)	Transpiration rate dependent of radiation energy available	Hour	VPD-limited transpiration model
*T*_*v*_(*t*)	Transpiration rate dependent on vapor pressure deficit (VPD)	Hour	VPD-limited transpiration model
*T*(*t*)	Transpiration rate dependent on radiation energy available, VPD, and soil water available.	Hour	VPD-limited transpiration model
*W*_*demand*_(*t*)	Cumulative soil water demand from plant from sunrise, *t*_*sunrise*_, to evaluated *t*. *W*(*t*_*sunset*_) is the daily soil water demand that is passed back to the water class in APSIM's daily process.	Hour	VPD-limited transpiration model
*B*(*t*)	Cumulative biomass accumulated by plant from sunrise, *t*_*sunrise*_, to evaluated *t*. *B*(*t*_*sunset*_) is the daily biomass that is passed back to the biomass class in APSIM's daily process.	Hour	VPD-limited transpiration model

*The time-scale column describes the time-scale (e.g., development stage, day, or hour) on which the variable changes or is evaluated. The origin column describes from where the variable is initialized or calculated (e.g., from a sub-class of the APSIM sorghum module, from the implementation of the VPD-limited transpiration model, or parameterized from experimental data)*.

Transpiration rate dependent on radiation energy available,

$$\begin{array}{l} T_{r}\left(t\right)=B_{r}\left(t\right)÷\frac{{TE}_{c}}{vpd\left(t\right)}÷lai. \end{array}$$

Transpiration rate dependent on VPD,

$$\begin{array}{l} T_{v}(t)=(\left(m_{T}(vpd(t))\times vpd\left(t\right)\right)\\ \;+\left(T\left(vpd_{BP}\right)-\left(m_{T}(vpd(t))\times vpd_{BP}\right)\right)), \end{array}$$

where $m_{T}(vpd(t))\text{=}\{\begin{array}{c} \text{​​}m_{1}\;vpd(t)<vpd_{BP}\\ m_{2}\;vpd(t)\geq vpd_{BP} \end{array}$, and *T*_*v*_(*t*) ≥ 0∀*t*, and *T*(*vpd*_*BP*_) = *m*_1_ × *vpd*_*BP*_ − (−(1/4) × *m*_1_) so that at VPD less than or equal to (1/4) there is no transpiration (Gholipoor et al., 2010).

Transpiration rate dependent on VPD, radiation energy available, and extractable soil water,

$$\begin{array}{l} T\left(t\right)=\min\left(T_{v}\left(t\right),\;T_{r}\left(t\right),W_{soil}\right). \end{array}$$

Then, for every day time hour, daily soil water used is calculated as the sum of the hourly products of transpiration rate by leaf area, *T*(*t*) × *lai*,

(1) $$\begin{array}{r} W_{demand}\left(t\right)=lai\;\times \sum_{i=t_{sunrise}}^{t}T\left(i\right) \end{array}$$

And, daily biomass is calculated as the sum of the hourly product of transpiration rate, *T*(*t*), leaf area, *lai*, and transpiration efficiency, $\frac{TE_{c}}{vpd\left(t\right)}$.

(2) $$\begin{array}{r} B\left(t\right)\;=\;lai\;\times TE_{c}\times \sum_{i=t_{sunrise}}^{t}\frac{T\left(i\right)}{vpd\left(i\right)} \end{array}$$

### Data and code accessibility

The files used to simulate energy sorghum crop growth and the code to modify growth based on VPD-limited transpiration can be found on GitHub (www.github.com/MulletLab/sorghum-energy-crop-model/).

## Results

### Modeling bioenergy sorghum in APSIM

To extend the applicability of APSIM to bioenergy sorghum, parameters of a sorghum genotype distributed with APSIM were modified to simulate bioenergy sorghum characteristics, namely an extended period of vegetative growth, high leaf area index (LAI), leaf number, radiation use efficiency, height, and stem density (Table 1). The sorghum model parameters in APSIM were adjusted based on traits measured as part of previous field studies conducted in 2008 and 2009 that characterized the growth and development of the energy sorghum hybrid, TX08001 near College Station, TX (Olson et al., 2012). In the prior field studies, data was collected on the energy sorghum hybrid TX08001 grown using three irrigation regimes: a rainfed environment with no irrigation, a limited irrigation scheme where plants were watered as needed until July 7th (applied in 2008 and 2009) and an unlimited irrigation scheme where plants were watered throughout the season (applied in 2009). Above ground biomass was measured in both years, and in 2008, data on stem height, leaf number, and leaf area index were collected. After parameterization based on 2008 trait data, APSIM simulations were compared to other years and irrigation schemes.

**Figure 1:**
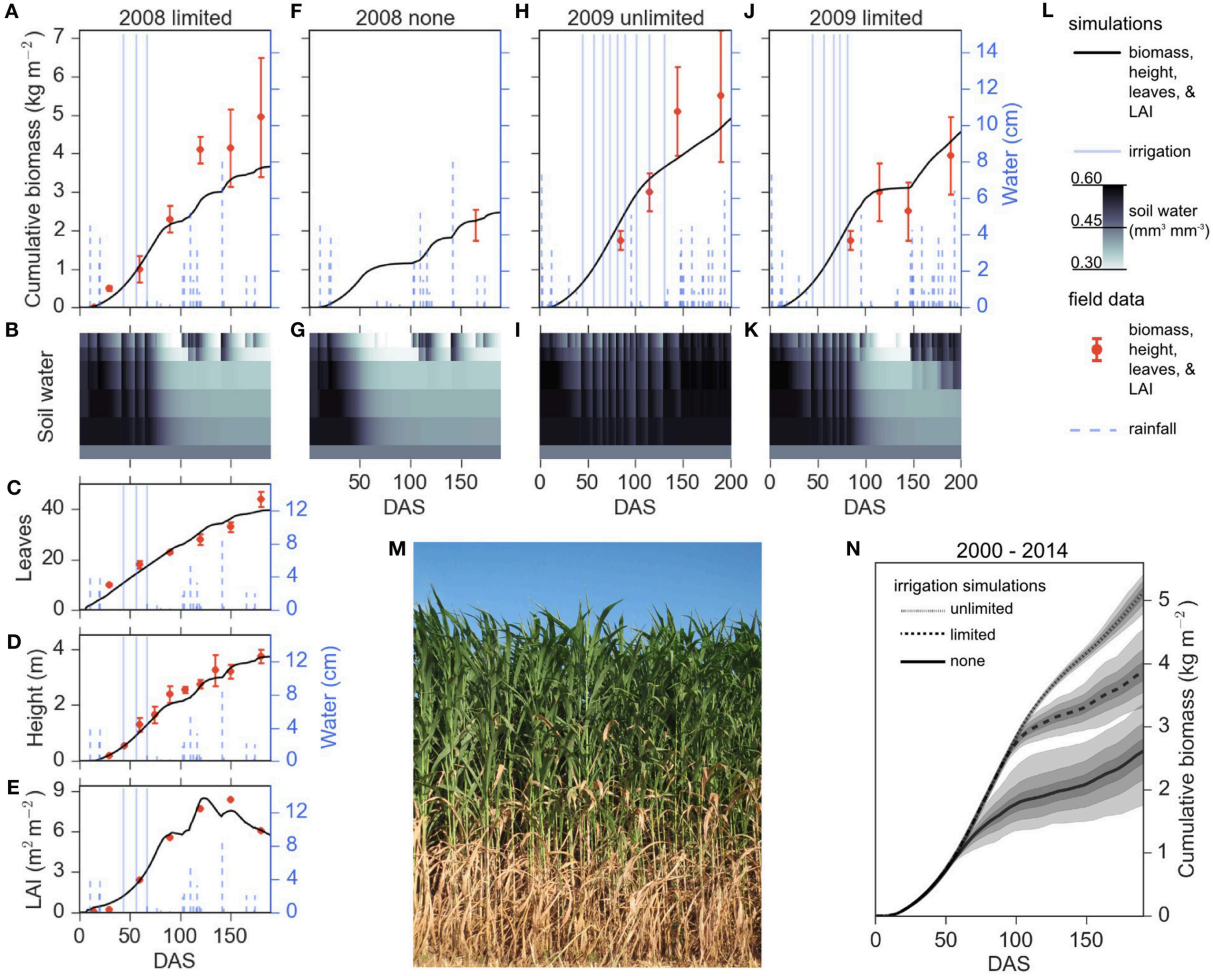
**Growth simulations and field data of energy sorghum in unlimited, limited, and no irrigation regimes**. Predicted biomass accumulation given 2009 and 2008 environmental conditions under unlimited **(H)**, limited **(A, J)**, and no **(F)** irrigation regimes fall within the margin of measurement error. Predicted leaf number **(C)**, plant height **(D)**, and leaf area index (LAI) **(E)** agree with experimental observations. Simulated water profiles from the three irrigation conditions are illustrated **(B,G,I,K)**. The respective soil water layers 1–6 are illustrated where the layers are 0–150, 150–300, 300–600, 600–900, 900–1200, and 1200–1500 mm depth, respectively. Plotting descriptors are provided in panel (**L**), and experimental data are plotted showing means and error bars representing one standard deviation where available, and the minimum and maximum range of observations in **F** (Olson et al., 2012). **(N)** Average simulated biomass accumulation for the 2000–2014 cropping seasons for different irrigation regimes. The means of unlimited, limited, and no irrigation are plotted with continuous, dashed, and dotted lines, respectively. Each irrigation regime has 68, 98, and 100 percent confidence intervals depicted with decreasing shades of gray. The mean and confidence intervals were estimated with 50,000 bootstraps. **(M)** An image of energy sorghum hybrid, TX08001, in 2016 College Station, TX, cropping season 108 days after sowing.

Growth simulations of energy sorghum for the unlimited, limited, and no irrigation conditions were qualitatively consistent with field data (Figure 1). A photograph of typical field plot of TX08001 at 108 DAS grown with limited irrigation is shown in Figure 1M. The front of the plot has been removed revealing the onset of leaf senescence at lower levels of the canopy that is associated with nitrogen recycling to support production of new leaves at the top of the canopy (Olson et al., 2012, 2013). The model largely tracked LAI, height, and number of leaves produced during the 2008 limited irrigation season (Figures 1C–E). The energy sorghum model also qualitatively reproduced the biomass accumulation trajectories observed in the limited and rainfed plots of 2008 and 2009. In 2008 and 2009, limited irrigation plots produced approximately 4 kg m^−2^ (~40 Mg hectare^−1^) of dry shoot biomass and unlimited irrigation plots in 2009 produced ~5 kg m^−2^ (~50 Mg hectare^−1^) of dry shoot biomass. Model predictions of biomass accumulation in 2009 with unlimited irrigation tracked field data for most of the season but predicted somewhat lower biomass accumulation late in the growing season, a trend also observed when modeling biomass accumulation in 2008 under limited irrigation. In the targeted management regimes of energy sorghum, under strictly rainfed conditions, the bioenergy sorghum crop model predicted biomass accumulation that was within the range observed in 2008 mechanical harvests of larger plots (Olson et al., 2012). These results indicate that APSIM is capable of predicting growth and biomass accumulation trajectories observed in field experiments within the margin of uncertainty of available data on field grown bioenergy sorghum across multiple years and water supply conditions.

The parameterized APSIM energy sorghum model was used to simulate how an energy sorghum crop would perform in the College Station, Texas, environment under different irrigation regimes and rainfall patterns over the annual growing seasons from 2000 to 2014 (Figure 1N). Using rainfall data from 2000 to 2014 in College Station, three water input regimes were imposed: unlimited, limited, and no irrigation (rainfed). As expected, the crop's ability to accumulate biomass in the three treatments diverges between 60 and 100 DAS when water becomes limiting, that is, when water in the initially saturated soil profile is depleted (Figures 1 B,G,I,K). Modeling showed that energy sorghum's long duration of vegetative growth allowed water capture from 100 DAS to 200 DAS and improved shoot biomass yield by ~30% in the water-limited production region relative to a crop harvested at 100 DAS.

**Figure 2:**
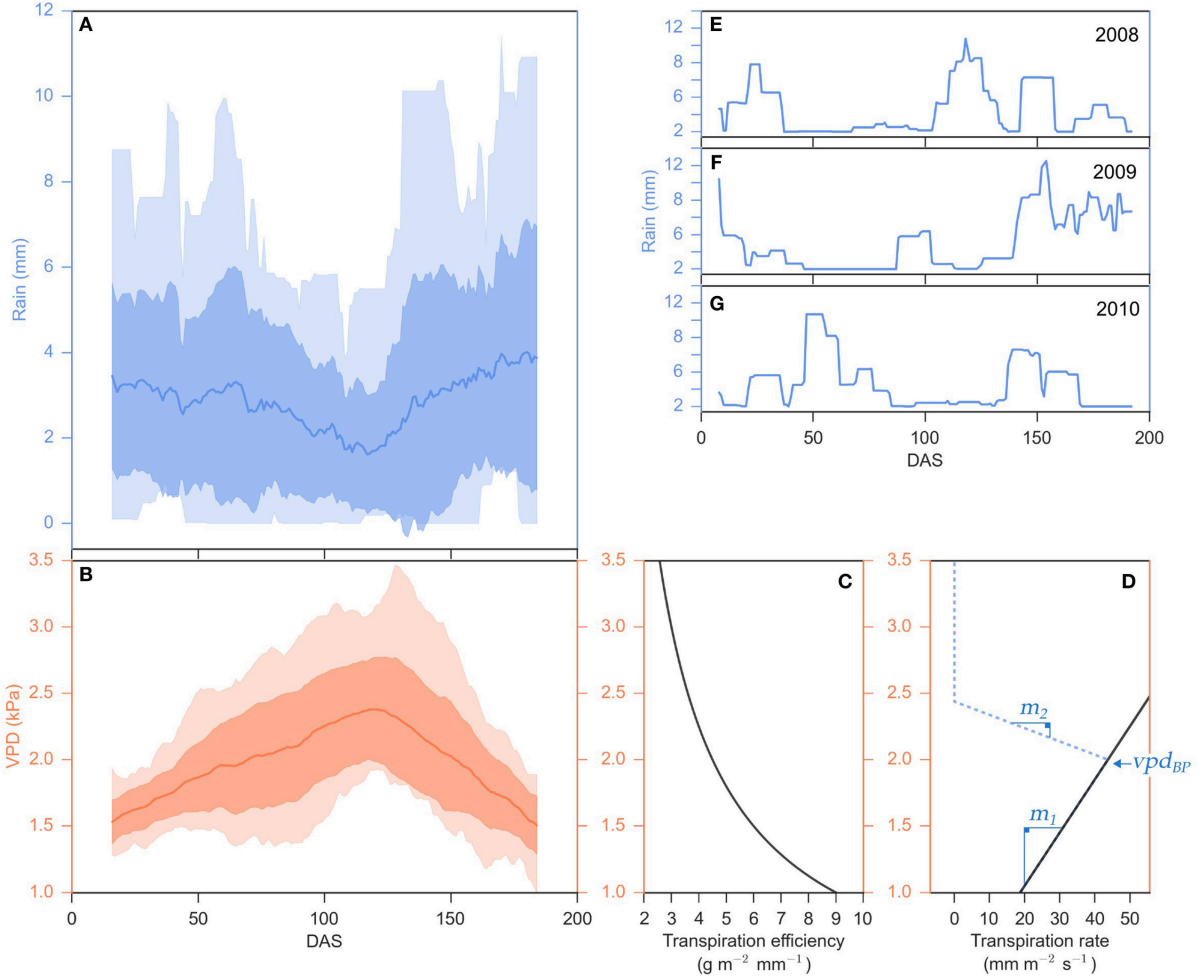
**Seasonal fluctuations of rainfall and VPD and their relationship to sorghum transpiration rate and efficiency. (A,B)** Distribution of daily rainfall (mm) and daily vapor pressure deficit, VPD, (kPa) over the 2000–2014 cropping seasons in College Station, TX, calculated as a mean of a 30-day sliding window. The mean is plotted as a solid line, the lighter transparent fill is the entire data range (minimums and maximums) and the darker transparent fill is one standard deviation from the mean. **(E–G)** Distribution of daily rainfall (mm) for the 2008 **(E)**, 2009 **(F)**, and 2010 **(G)** cropping seasons in College Station, TX, calculated as a mean of a 14-day sliding window. **(C)** Transpiration efficiency, biomass produced per unit of water transpired (g m^−2^ mm^−2^), and **(D)** a hypothetical transpiration rate (solid black line), the amount of water uptake per unit time (mm m^−2^ s^−2^), and their responses to VPD (kPa) are plotted along the x-axes sharing the y-axis of panel (**B**). VPD-limited transpiration trait parameters {*vpd*_*BP*_, *m*_1_, *m*_2_} is denoted in blue.

Fluctuations in rainfall and VPD during the 2000–2014 growing seasons in College Station, Texas are shown in Figure 2. The amount and timing of rainfall was highly variable but generally decreased from planting to ~120 DAS (mid-summer) and then increased again during the latter portion of the growing season (Figure 2A). Profiles of rainfall patterns in 2008, 2009, and 2010 show that rainfall between 75 and 150 DAS is intermittent and insufficient to recharge the soil profile (Figures 2E–G). In the location modeled, average daily VPD increased from planting until ~120 DAS then declined until harvest at ~200 DAS (Figure 2B). VPD also fluctuates significantly on a daily basis (Supplemental Figure 1). Increases in VPD generally result in higher rates of transpiration and water uptake from the soil profile by the root system, and lower efficiency of conversion of atmospheric carbon to plant biomass per unit of water transpired (TE) (Figures 2C,D). In simulations where water is not a limiting factor (unlimited irrigation) and transpiration efficiency is not influencing biomass yield, energy sorghum is predicted to yield 5.4 ± 0.3 (mean ± SD) kg m^−2^ of biomass while using 1132 ± 82 (mean ± SD) mm m^−2^ of water during a 200 day growing season in College Station cropping environments. In contrast, in the absence of irrigation, energy sorghum was predicted to produce on average 2.7 ± 0.8 (mean ± SD) kg m^−2^ of biomass and used 485 ± 120 (mean ± SD) mm m^−2^ of water (Figure 1N). The results of modeling indicate that water supply significantly limits sorghum biomass accumulation, especially after 60–75 DAS, in this location consistent with field observations (Olson et al., 2012; Gill et al., 2014; Mullet et al., 2014).

### Modeling VPD-limited transpiration traits

Transpiration efficiency (TE) decreases with increasing VPD (Hammer et al., 1997). Therefore, the APSIM sorghum model was used to examine the potential utility of traits that limit transpiration at higher VPD for improving the biomass yield, water use efficiency, and resilience of bioenergy sorghum. Previous experimental work demonstrated that VPD-limited transpiration traits are dependent on VPD and sensitive to 1 kPA changes in VPD (Gholipoor et al., 2010; Choudhary et al., 2013; Choudhary and Sinclair, 2014b; Riar et al., 2015). Energy sorghum crops are exposed to a range of VPDs greater than 1 kPA over the course of a day due to daily variation in temperature (Supplemental Figure 1). Therefore, the APSIM model was modified so that the calculated daily water uptake could be altered by a VPD-limited transpiration trait calculated in hourly timesteps, an approach similar to that implemented by Sinclair et al. (2005), Kholová et al. (2014), and Messina et al. (2015). VPD-limited transpiration traits were modeled using two different linear response slopes, *m*_1_ and *m*_2_ where the transition between *m*_1_ and *m*_2_ is determined by a VPD threshold, *vpd*_*BP*_ (Figure 2D, denoted in blue). The potential utility of genotypes with variation in *m*_1_ or *m*_2_, or combinations of VPD-breakpoints and values of *m*_2_ was examined. Modeling enabled the impact of various combinations of VPD-traits on the time course of biomass accumulation, LAI development and soil water extraction dynamics to be investigated.

### Modeling genotypes that vary in *m*_1_ that lack VPD-breakpoints

**Figure 3:**
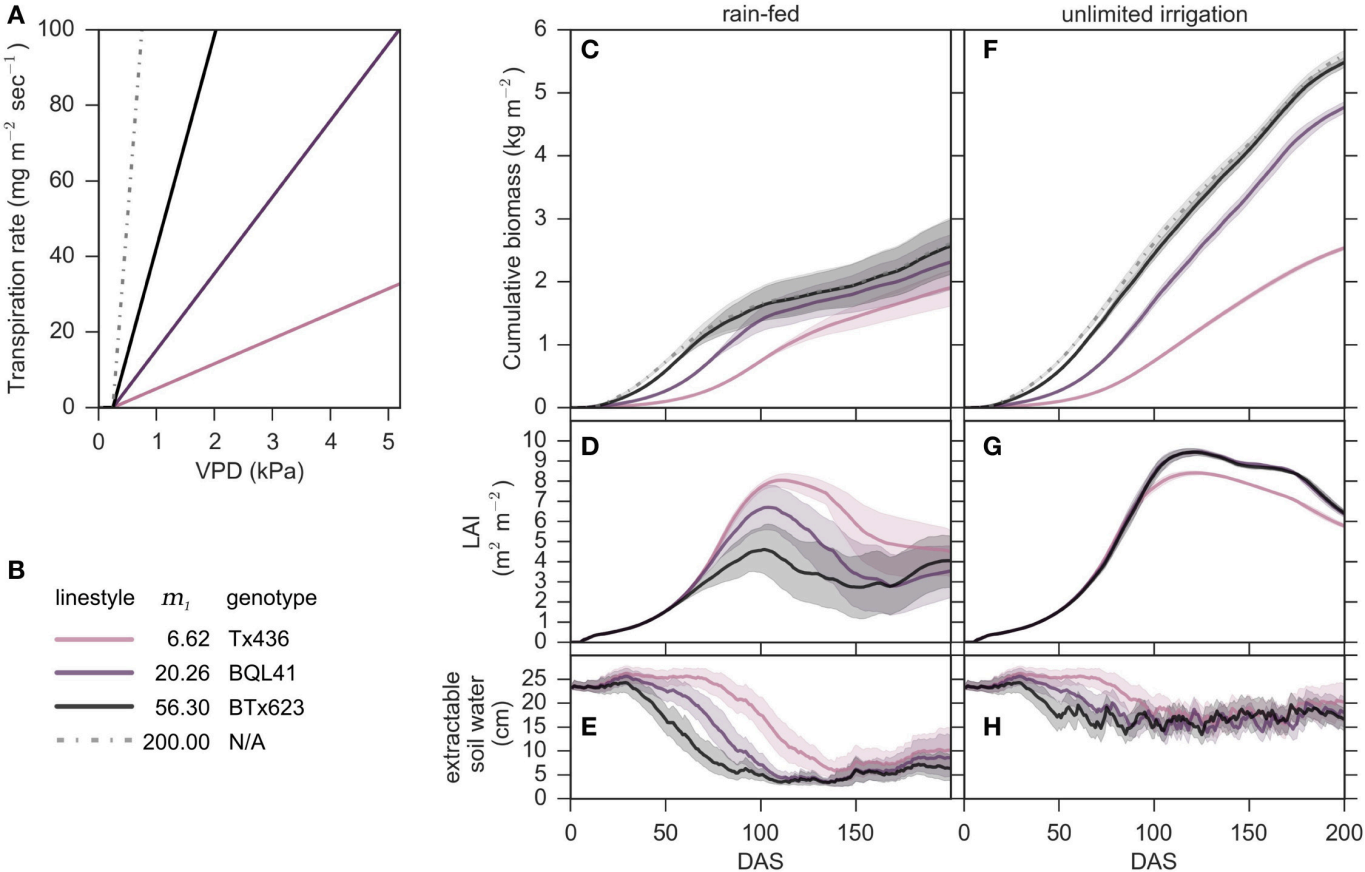
**Energy sorghum biomass accumulation from 2000 to 2014 cropping seasons simulated in College Station, TX, with water supply regimes of unlimited irrigation and only rainfed. (A–H)** Growth simulation of plants with no *vpd*_*BP*_ (*m*_1_ = *m*_2_) are plotted with continuous colored lines that correspond to their *m*_1_ ∈ {6.62, 20.26, 56.3, 200}. **(C,F)** Cumulative biomass, **(D,G)** leaf area index (LAI), and **(E,H)** extractable soil water are plotted as the means and 98% confidence intervals estimated with 50,000 bootstraps for the respective rainfed and unlimited irrigation conditions.

Modeling was used initially to investigate genotypes that vary in *m*_1_ slopes that lack a *vpd*_*BP*_ (Figure 3). Specifically, Tx436 (*m*_1_ = 6.62; Choudhary et al., 2013), BQL41 (*m*_1_ = 20.26; Choudhary et al., 2013), BTx623 (*m*_1_ = 56.3; Gholipoor et al., 2010) and a hypothetical extreme (*m*_1_ = 200.0) were simulated using the VPD-limited transpiration modification with no *vpd*_*BP*_ (*m*_1_ = *m*_2_) (Figure 3A). In fully irrigated conditions, genotypes with higher values of *m*_1_ accumulated more biomass (Figure 3F). A modeled genotype similar to BTx623 (*m*_1_ = 56.3) accumulated almost twice as much biomass (an additional ~26 Mg/ha) compared to Tx436 (*m*_1_ = 6.62) under these conditions (Figure 3F). Values of *m*_1_ > 56.3 did not increase end-point biomass accumulation. Interestingly, the genotype with an *m*_1_ = 20.26 (BQL41) accumulated less biomass during the first 75 days of development in fully irrigated conditions compared to BTx623 (*m*_1_ = 56.3), however biomass accumulation by the two genotypes was similar between 75 and 200 DAS. This occurred because later in the season when the genotypes reached canopy closure and VPD was higher, both genotypes extracted the maximum available soil water each day.

In rainfed cropping conditions a genotype with an *m*_1_ of 56.3 was predicted to yield approximately 2.5 kg m^−2^ approximately 50% less biomass than in non-water limiting conditions (Figure 3C). In rainfed environments, increased values of *m*_1_ on average also resulted in higher biomass accumulation, however, the benefit of a higher *m*_1_ in water limited environments was much smaller than in fully irrigated conditions (~1–3 Mg/ha) (Figure 3C). While biomass accumulation was positively related to larger *m*_1_ values, it came at the expense of leaf area development and maintenance under water limiting conditions (Figure 3D). During the first 60–75 days of crop development, genotypes with larger *m*_1_ (BTx623) accumulated biomass more rapidly in rainfed and fully irrigated environments compared to genotypes with lower *m*_1_ values. The rate of LAI increase was similar in all genotypes until ~60 DAS because leaf area development in all genotypes was limited by thermal time in the model. Genotypes with larger *m*_1_ values use water more rapidly therefore by 60 DAS the genotype with an *m*_1_ = 56.3 (BTx623) had depleted ~50% of available soil water in rainfed environments, more than the other genotypes (Figure 3E). For a genotype similar to BTx623, lack of sufficient water supply in rainfed environments constrained biomass accumulation and leaf area development for the remainder of the season (Figures 3C, D). Compared to a genotype with a genotype with an *m*_1_ = 56.3, genotypes with lower *m*_1_ values used water more slowly and accumulated less biomass in rainfed and fully irrigated conditions (Figures 3C,F). In rainfed conditions, slower use of water provided additional thermal time for canopy development before soil water was depleted resulting in higher LAI (Figure 3D). In rainfed environments, modeling predicted that LAI would decrease once soil water resources were expended beginning about 100 DAS (Figures 3D,E). LAI declined sooner in genotypes with higher *m*_1_ because they used up water resources more rapidly. Despite lower leaf area after 150 days in rainfed environments, genotypes with higher *m*_1_ (56.3, 20.26) accumulated similar amounts of biomass between 150 and 200 DAS on average over the 14 years analyzed as the genotype with a lower *m*_1_ (Tx436, *m*_1_ = 6.62)

### Modeling energy sorghum genotypes with VPD-breakpoints and variation in *m*_2_

The VPD-limited transpiration trait model was next used to model energy sorghum genotypes that regulate transpiration using a *vpd*_*BP*_ where the slope of the transpiration rate response to VPD changes from *m*_1_ to *m*_2_. BQL41 is characterized by a low *vpd*_*BP*_ of 1.17 kPA and the genotype's transpiration response to VPD is postulated to improve performance of grain sorghum in water limited environments (Choudhary et al., 2013). To examine the physiological effect of BQL41's low *vpd*_*BP*_ on biomass yield and water use efficiency of bioenergy sorghum in water-limited environments, biomass accumulation of a VPD-limited transpiration trait that reflects the *m*_1_ and *vpd*_*BP*_ of BQL41 was combined with variation in *m*_2_ values. Five *m*_2_ values were evaluated: (a) *m*_2_ = 7.27, the transpiration rate that was experimentally obtained from the BQL41 genotype, (b) *m*_2_ = 0.0, a maximum transpiration rate which reflects how VPD-limited transpiration was modeled previously (Sinclair et al., 2005; Kholová et al., 2014; Messina et al., 2015), (c) *m*_2_ = 20.26, a response that reflects a lack of a *vpd*_*BP*_ (i.e., *m*_1_ = *m*_2_ = 20.26), (d) *m*_2_ = −6.1, and (e) *m*_2_ = −12.49 transpiration responses to VPD that were experimentally obtained from SC803 and SC35 genotypes by Gholipoor et al. (2010) and Choudhary et al. (2013), respectively (Table 3). In rainfed College Station environments the total biomass accumulated by each of the genotypes with VPD-breakpoints investigated ranked from largest to smallest was: 2.35 kg m^−2^ (SC803; *m*_2_ = −6.1), 2.31 kg m^−2^ (no *vpd*_*BP*_; *m*_2_ = 20.26), 2.29 kg m^−2^ (maximum limited transpiration; *m*_2_ = 0), 2.28 kg m^−2^ (BQL41; *m*_2_ = 7.27), and 2.25 kg m^−2^ (SC35; *m*_2_ = −12.49) (Supplemental Table 1). In these simulations, SC803 (*m*_2_ = −6.1) marginally outperformed the genotype lacking a *vpd*_*BP*_ over a 14 year average by 1.6 % (Supplemental Table 1).

**Table 3:** **VPD-limited transpiration parameters { *m*_1_, *m*_2_, *vpd*_*BP*_ } of genotypes used in this study**.

**Genotype**	***m*****_1_**	***m*****_2_**	***vpd*****_*BP*_**	**Literature**
BQL41	20.26	7.27	1.17	Choudhary et al., 2013
BTx623	56.3	10.4	2.05	Gholipoor et al., 2010
SC803	39.8	−6.1	2.29	Gholipoor et al., 2010
SC35	8.96	−12.49	2.91	Choudhary et al., 2013
Tx436	6.62	−	−	Choudhary et al., 2013

*These values were experimentally obtained from their respective citations*.

The relatively small improvement in biomass yield in genotypes with VPD-breakpoints was further analyzed to identify trade-offs that occur over the course of the growing season in the rainfed environments of College Station, TX (Supplemental Figure 2). The genotypes with a *vpd*_*BP*_ have slower growth and biomass accumulation at the beginning of the season (through 100 DAS) and accumulate more biomass during the latter part of the season (Supplemental Figure 3D). This shift in the timing of water use from early in the season to the summer months when VPD is higher may contribute to lower biomass yield even though the trait partially mitigates the influence of higher VPD. Genotypes with the lower *m*_2_ reach a higher LAI between 100 and 150 DAS than the genotypes with the higher *m*_2_ (Supplemental Figure 2). This trend in genotypes with higher *m*_2_ is correlated with a reduced rate of depletion of extractable soil water that allows for a longer period of canopy production (Supplemental Figure 2F). Most genotypes show a significant loss of LAI once soil water supply is depleted beginning between 100 and 150 DAS depending on genotype (Supplemental Figures 2E,F). Genotypes with a low *m*_2_ such as SC35 maintain higher LAI for the remainder of the growing season (Supplemental Figure 2E).

Since the VPD-limited transpiration trait has a negative impact on biomass accumulation during the early portion of the season, but a potentially beneficial impact during the summer months, a simulation was run where the trait was induced after 75 DAS in a terminal drought environment to better understand the dynamics involved (Supplemental Figure 3). In this simulation, the VPD-limited transpiration trait reduced the rate of water utilization, delayed the onset of leaf senescence, and increased water use efficiency and biomass accumulation. A genotype with no breakpoint accumulated 0.85 kg m^−2^, whereas genotypes with VPD-limited transpiration accumulated 1.2 kg m^−2^ between 75 DAS until all soil water was extracted. Simulations showed that induction of the VPD-limited transpiration trait under well watered conditions reduced biomass yield (Supplemental Figure 3F).

### Integrated modeling of VPD-traits

**Figure 4:**
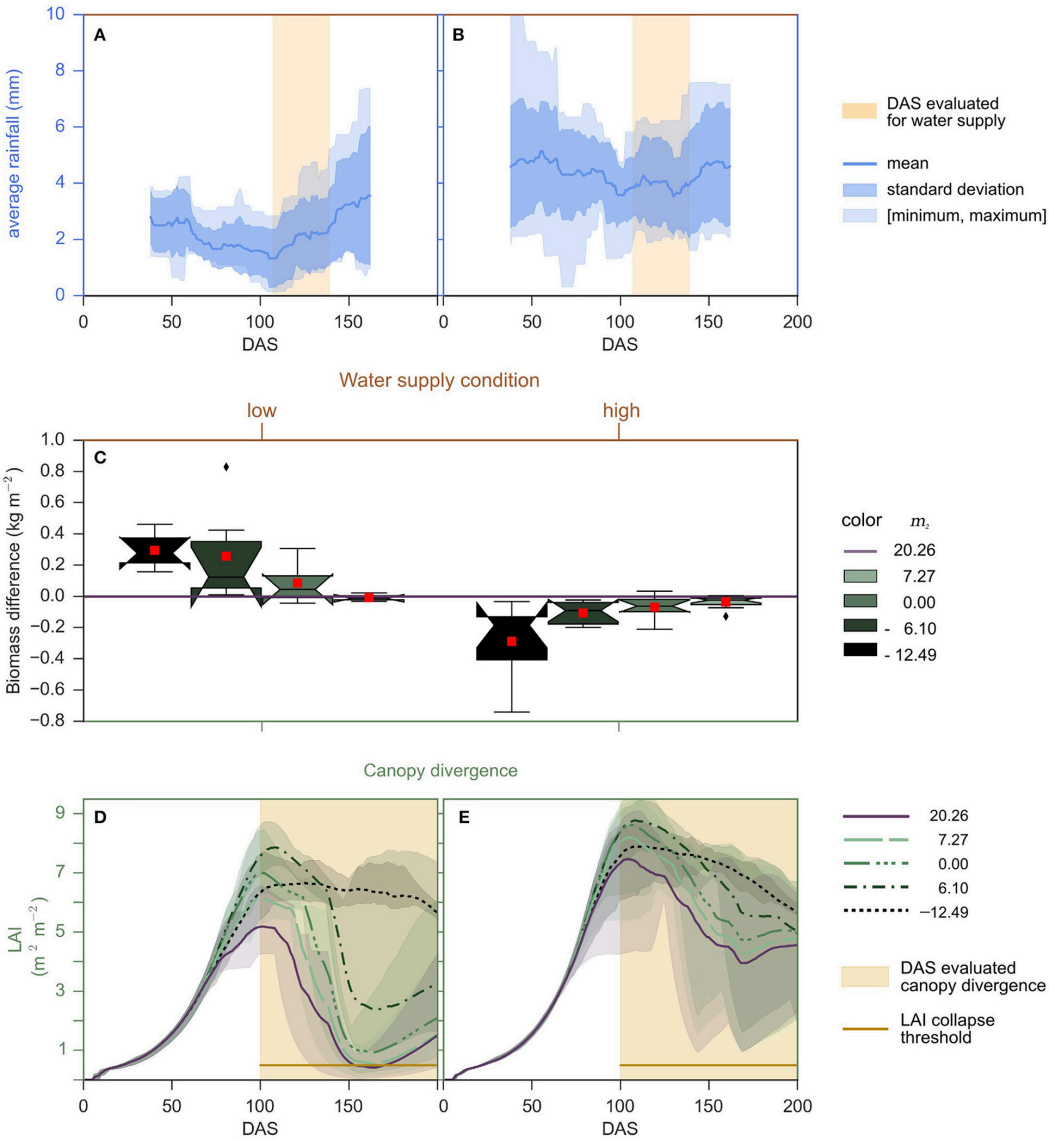
**Performance of genotypes with VPD-limited transpiration traits determined in years with low and higher rainfall from 107 to 139 DAS and determined by canopy maintenance. (C)** Boxplots show the biomass difference across 14 years between genotypes with a VPD-limited transpiration trait and the genotype that lacks a *vpd*_*BP*_ (*m*_2_ = 20.26). The notches represent the 95% confidence interval of the median. The red square represents the means. These statistics are plotted against end biomass accumulated (DAS 200) by the genotype that lacks a *vpd*_*BP*_, where the vertical sets of plots are separated by years categorized on the **(A,B)** water supply condition and **(D,E)** canopy divergence condition. **(A,B)** For the drought condition, rainfall per day is calculated from sliding windows of 75 days. For sliding windows that center from DAS 107 to 139, if the average rainfall per day ever falls below 1.2 mm, then the year is considered a drought year. The mean, standard deviation, and entire range of rainfall in sliding windows for the years considered low and high are plotted, respectively. The center of sliding windows evaluated for the water supply condition is highlighted orange. **(D,E)** For the canopy divergence condition, where the vertical sets of plots are separated by the condition that occurs in the latter half of the cropping season. For each year, for each pairwise comparison between a genotype with and without a *vpd*_*BP*_, and for each day in the latter half of the cropping season (≥ 100 DAS), if the canopy of genotype lacking a *vpd*_*BP*_ senesced (< 0.5 LAI) and the genotype with a *vpd*_*BP*_ maintained a larger canopy, then VPD-limited transpiration genotype (with a *vpd*_*BP*_) was predicted to out perform the genotype lacking a *vpd*_*BP*_. LAI is plotted as averages of the conditions met and the 12.5–87.5 inter-percentile range.

The differences in biomass accumulation relative to timing of water limitation indicates that the benefits of the VPD-limited transpiration trait are greater when VPD is high and water supply is limiting during the cropping season. To examine this dynamic further, years in the College Station cropping environment were identified where the average rainfall per day fell below 1.2 mm between DAS 107–139 given a 75-day sliding window average (Figures 4A,B). On average, years with lower rainfall from 107 to 139 DAS also had lower total rainfall and higher average VPD (Supplemental Table 1). Applying this criterion to rainfed cropping environments of College Station, TX, for 2000—2014, and then evaluating the previous combination of *m*_1_, *m*_2_, and *vpd*_*BP*_ indicated that the VPD-limited transpiration trait is beneficial in the more water-limited environments, since VPD-limited transpiration plants with negative *m*_2_ parameters always yielded greater biomass (Figures 4A–C, Supplemental Table 1). For example, the sorghum genotype with maximum limited-transpiration (*m*_2_ = 0) on average yielded more biomass than a sorghum genotype lacking a *vpd*_*BP*_ (*m*_2_ = *m*_1_ = 20.26) in the water-limited low yield environments (Supplemental Figure 4). Moreover, differences in predicted canopy maintenance was correlated with, and may help explain why VPD-limited transpiration plants outperform their non-limited transpiration counterparts (Figures 4D,E). In summary, more restrictive transpiration responses associated with more negative *m*_2_ values perform progressively better in water-limited, low-yielding environments that lead to loss of canopy, but progressively worse in higher rainfall conditions (Figures 4A,B).

**Figure 5:**
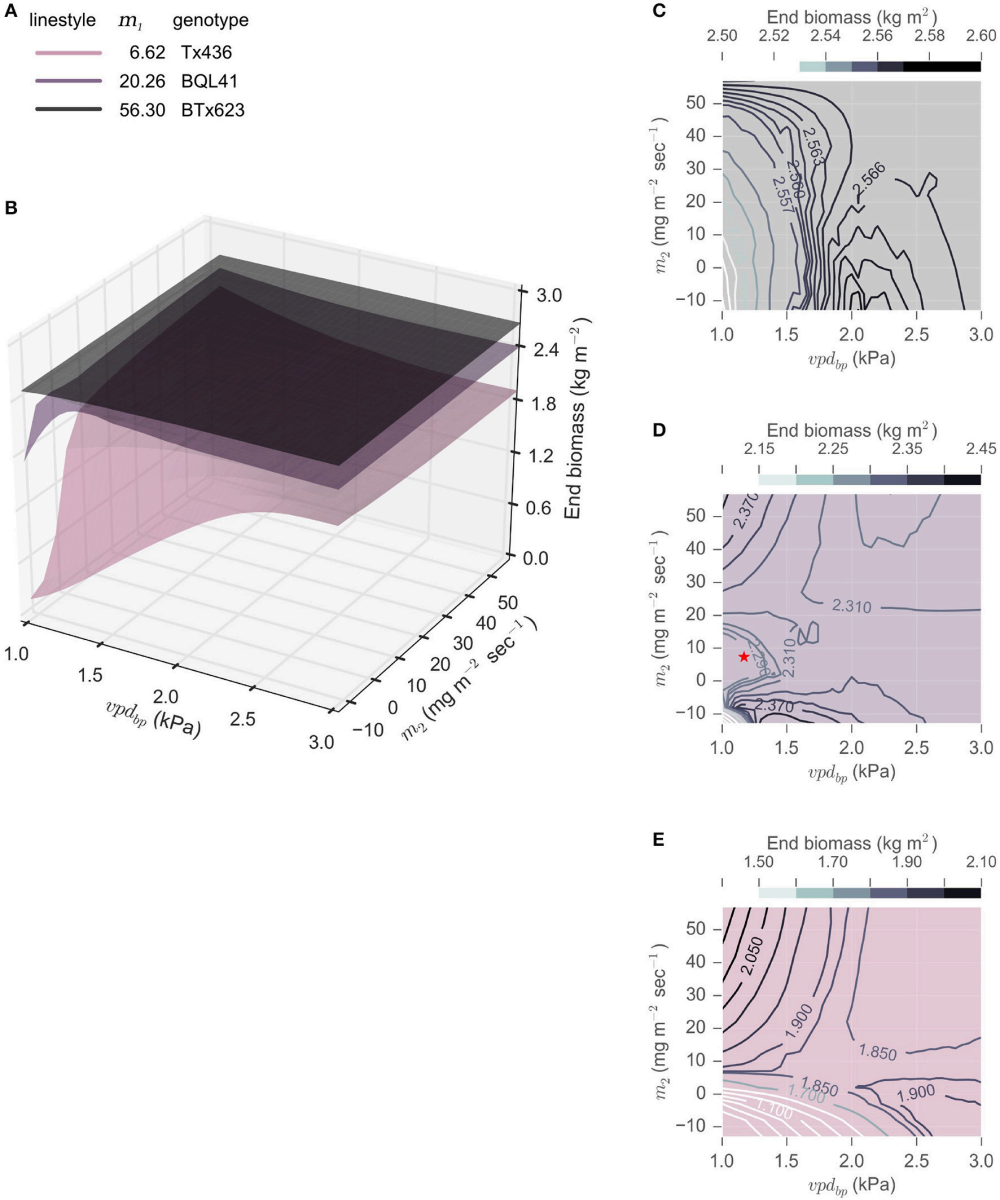
**Landscapes of energy sorghum productivity for varying *m*_1_, *m*_2_ and *vpd*_*BP*_ within the observed phenotypic parameters. End biomass at 200 DAS predicted from rainfed College Station, TX (2000 – 2014) was used as a measure of productivity. (A)** *m*_1_ was varied to reflect the extremes (6.62, Tx426, and 56.3, BTx623) and a moderate transpiration rate (20.26, BQL41). **(B)** *m*_2_ ∈ [−12.49, 56.3] and *vpd*_*BP*_ ∈ [1.17, 2.91] are evaluated for each *m*_1_ and the predicted average end biomass was interpolated to construct the topological surfaces. **(D–E)** The topology of each *m*_1_ surface is also plotted two-dimensionally, with contour lines to depict variation of end biomass at 200 DAS. The red star in panel (**D**) represents the VPD-limited transpiration parameters of BQL41.

To examine the influence of all three parameters describing VPD-limited transpiration, *vpd*_*BP*_ and *m*_2_ were varied within the genetic range observed to date for sorghum, and their impact on biomass yields given different *m*_1_ parameters were evaluated (*m*_1_ ∈ {6.62, 20.26, 56.3}). A range of *vpd*_*BP*_ values (*vpd*_*BP*_ ∈ [1.17, 2.91]) representing the range bounded by BQL41 and SC35, respectively, and *m*_2_ values (*m*_2_ ∈ [−12.49, 56.3]), the range bounded by SC35 and BTX623, respectively, were explored (Gholipoor et al., 2010; Choudhary et al., 2013). Combinations of these parameters were simulated in rainfed College Station, TX, environments of 2000—2014 cropping seasons (Figure 5). The total biomass accumulated at 200 DAS was used as a measurement of seasonal crop productivity. These productivity landscapes illustrate the influence of VPD-limited transpiration traits, and indicate that, on average, in the target environment of College Station, TX, *m*_1_ is the greatest driver of average productivity of the sorghum crop. All simulated genotypes with an *m*_1_ = 56.3 produced more biomass than those with an *m*_1_ = 20.26 or 6.62, regardless of *m*_2_ and *vpd*_*BP*_. Contour plots of the three *m*_1_ values show that the extent of phenotypic variation introduced by the other two parameters *m*_2_ and *vpd*_*BP*_ is influenced by the magnitude of *m*_1_, where the variation in cumulative biomass increases as *m*_1_ decreases.

**Figure 6:**
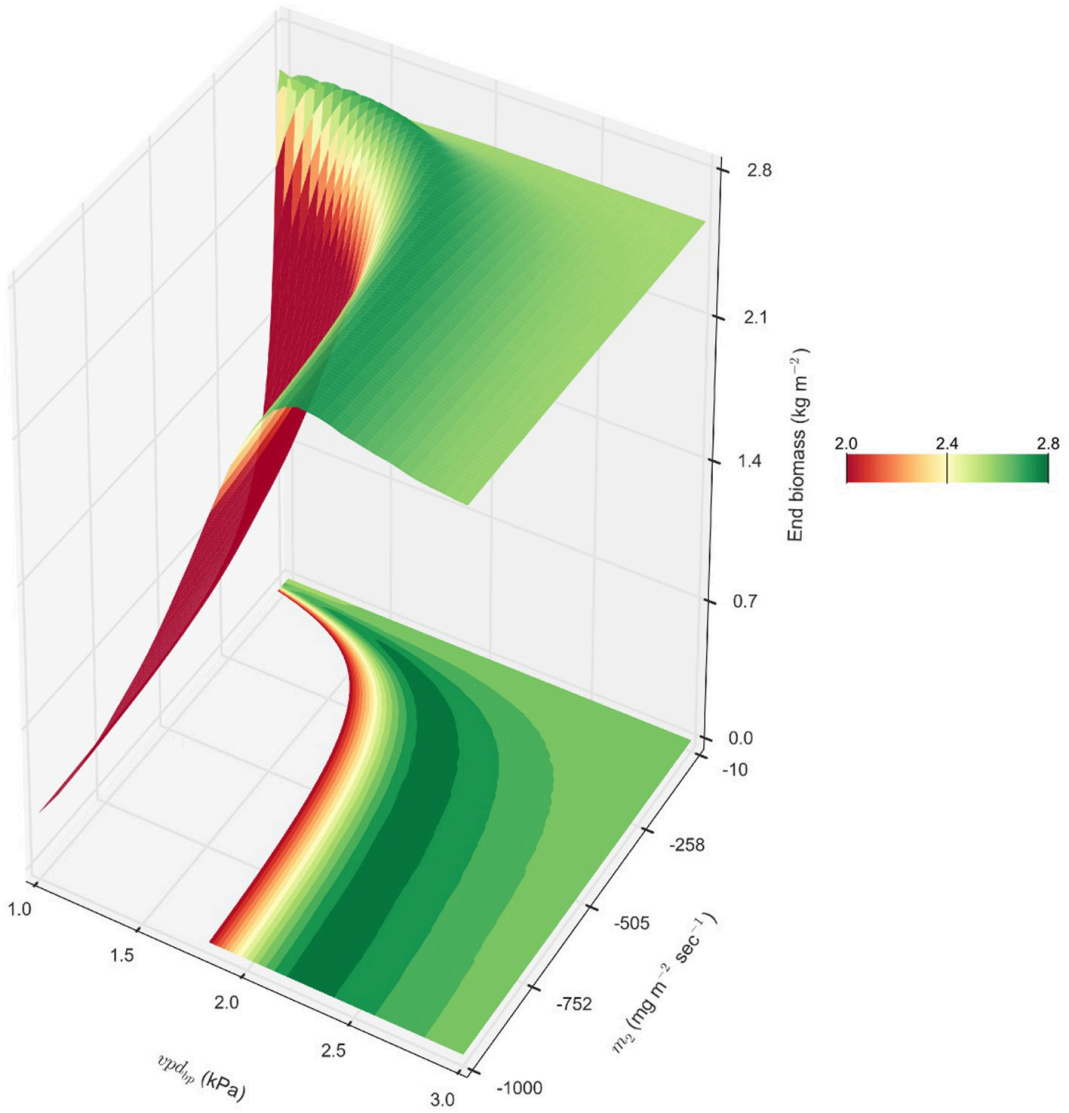
**Theoretical energy sorghum productivity landscape**. A combination of *m*_1_ = 56.3 representing BTx623, a range of *vpd*_*BP*_ values (*vpd*_*BP*_ ∈ [1.17, 2.91]) representing BQL41 and SC35, and a range of *m*_2_ values (*m*_2_ ∈ [−1000, −10]) beyond the observed genotypic parameters were evaluated for end biomass in rainfed College Station, TX, cropping environments 2000 – 2014, and their averages are interpolated to create the topology of end productivity.

The results of modeling show that genotypes with a high *m*_1_ (~56) accumulate more biomass when soil water resources are not limiting during the early portion of the season, and that the VPD-breakpoint trait is beneficial when water is limiting during the middle of the growing season when VPD is higher. There may exist a theoretical *m*_2_ for *m*_1_ = 56.3 that would be predicted to increase productivity beyond the 0.2% in the observed parameter space. For an *m*_1_ = 56.3, the exploration of a theoretical *m*_2_ outside what has been previously observed for VPD-limited-transpiration predicted that an *m*_2_ = −290 with a *vpd*_*BP*_ = 2.05 would increase biomass by 6.5%, yielding an average of 2.73 kg m^−2^ in rainfed College Station, TX (2000-2014) (Figure 6). This extreme *m*_2_ = −290 would be reflective of a genotype that essentially stops transpiration at VPDs higher than its *vpd*_*BP*_. Furthermore, observation of the fitness landscape suggests that it is not essential to find the exact combination of *vpd*_*BP*_ and *m*_2_, but that there is a gradual ridge constructed by combinations of the two VPD-limited transpiration parameters that would improve biomass accumulation by ≥5% (Figure 6). This ridge shows the relationship between the *vpd*_*BP*_ and *m*_2_ for increasing biomass by ≥5% in rainfed College Station, TX (2000–2014) in the VPD-limited transpiration diagram, where as *vpd*_*BP*_ increased, the range of *m*_2_ parameters that could be combined for beneficial productivity increases. This relationship with *vpd*_*BP*_ is likely related to the effect of VPD on transpiration efficiency, *TE*, which is inversely proportional to VPD through the *TE*_*c*_ coefficient: $TE=\frac{TE_{c}}{vpd}$.

## Discussion

Crop modeling and structure-function trait analyses can be used to help identify constraints that limit plant productivity, assess the benefits of traits, and to increase productivity by optimizing crop deployment across variable environments. Since energy sorghum is a relatively new C4 grass biomass crop with an unusually long vegetative growth phase, crop modeling could help identify traits useful to select in breeding programs. The APSIM bioenergy sorghum model developed in this study was able to replicate the developmental time course of leaf area development, leaf number, plant height and biomass accumulation observed in field trials in College Station, Texas. Field data from other locations/years and from newer energy sorghum hybrids would be useful to further validate and enhance the accuracy of modeling predictions. In addition, collection of detailed information on soil profiles, root system development, architecture, rates of soil water extraction and information on water deficit induced changes in ABA levels would further improve the energy sorghum model and to enable modeling of root traits in conjunction with canopy architecture and VPD-transpiration traits.

Model evaluation was done using the College Station, TX, cropping environment, a location with a long growing season (~200 days) and hot and dry summers where bioenergy sorghum hybrids are currently under selection in a breeding program. In fully irrigated conditions, modeling and field data show that energy sorghum has the genetic potential to accumulate ~50 Mg/hectare per year (Olson et al., 2012). However, due to sorghum's water use efficiency and drought resilience during the vegetative phase, it is likely that energy sorghum will often be grown in water limited environments less suitable for other crops. Under rainfed conditions where the median rainfall over the growing season used for the simulations was 52.3 cm, bioenergy sorghum is projected to yield approximately 25 Mg hectare^−1^ per year. This model prediction is consistent with the biomass yield of TX08001 (~15–26 Mg hectare^−1^) grown without irrigation in large plots that were machine harvested in 2008 and 2009 (Olson et al., 2012). Modeling of energy sorghum production in this location based on 2000–2014 environments indicated that energy sorghum would capture additional water resources and increase biomass yield by ~30% by extending the growing season from ~100–120 days typical of grain crops to 200 days. A crop designed to capture the additional water resources during a long growing season in this environment would benefit from high water use efficiency and resilience to water deficit. Sorghum exhibits many useful traits for water limited environments including high cuticular wax, deep rooting capacity, induction of leaf rolling and osmotic adjustment in response to water deficit, stay-green, and VPD-limited transpiration traits (Borrell et al., 2006). In this study the APSIM bioenergy sorghum model was used to examine the utility of VPD-limited transpiration traits by evaluating the impact of VPD modulated transpiration in hourly intervals during the day. The model extension was used to identify the benefits and trade-offs associated VPD-limited transpiration traits during growing seasons using College Station, TX environments as the context for this case study (mean ± SD: 56 ± 18 cm of rainfall per cropping season for 2000–2014).

Prior studies demonstrated that limited transpiration can result in improved grain yield in drought and heat stressed environments by shifting water utilization from the vegetative and booting phases to the anthesis and post anthesis phases where grain yield is more sensitive to water deficit (Sinclair et al., 2005; Kholová et al., 2014; Messina et al., 2015). This change in the timing of water use improves harvest index and reduces the risk of large reductions in grain associated with water limitation during the reproductive phase (Messina et al., 2015). The improvement is not attained if water is not sufficiently limited, an emergent property associated with limited transpiration traits in grain sorghum (Kholová et al., 2014; Hammer et al., 2016). We hypothesized that VPD-limited transpiration traits could also be beneficial for energy sorghum but for different reasons since the crop is harvested for biomass and grows most of the season in the vegetative phase. The analysis showed that restriction of transpiration by reducing *m*_1_ or by VPD-limited transpiration involving low VPD-breakpoints, reduced biomass accumulation early in the season and that this had a negative impact on overall biomass yield. There are several factors that could explain this prediction. First, at the start of the growing season the soil profile is saturated and excess rainfall is not captured due to run off until a portion of the soil water has been used for biomass production or is lost through evaporation. In this part of the season, modeling showed that genotypes with high *m*_1_ values like BTx623 that accumulate biomass and use water at a high rate outperform genotypes with lower *m*_1_ values. If a higher rate of biomass accumulation also results in more rapid leaf area development, then this would increase the efficiency of radiation interception during the phase of canopy development (0–60 DAS). However, the results showed that a higher biomass accumulation rate did not influence LAI development, a topic worth further investigation. Second, shifting water use for biomass accumulation from the early portion of the season (0–75 DAS) when average VPD is lower to later in the season (75–150 DAS) when VPD is higher and TE is lower reduces water use efficiency. The negative impact of this shift in the timing of water use can be mitigated in part by VPD-breakpoint limited transpiration traits. However, genotypes with current *vpd*_*BP*_ and *m*_2_ values still use water at higher VPD in the summer, although at lower rates, compared to water used at lower VPD during the first portion of the planting season. This interpretation is consistent with the predicted benefits of a theoretical *m*_2_ that completely shuts off transpiration above the *vpd*_*BP*_. In that hypothetical genotype, transpiration would be restricted to VPD below the breakpoint during the entire growing season mitigating the impact of shifting the seasonal timing of water use. Third, in high rainfall years, VPD-limited transpiration could reduce the use of all available water resources for biomass production, especially when significant rainfall occurs in the last 60 days of the growing season. These trade-offs may help explain why in optimization simulations (Figure 5), genotypes with higher *m*_1_ values and higher VPD-breakpoints (i.e., *m*_1_ = 56.3, *vpd*_*BP*_ = 2.05) were found to accumulate more biomass in the environments analyzed because these parameters allowed maximal biomass accumulation early in the growing season.

The value of *vpd*_*BP*_ and *m*_2_ traits that restrict use of water during portions of the day with high VPD and low TE for energy sorghum was evident during the summer months when VPD is high and water supply is limited. In a simulated summer terminal drought environment, genotypes with this type of VPD-limited transpiration trait accumulated more biomass compared to genotypes lacking the trait consistent with higher TE (Supplemental Figure 3). The trait also slowed the use of water and delayed the onset of leaf senescence that begins when most of the soil water supply has been depleted. The delay or reduction in the extent of leaf senescence during summers with low rainfall would improve the crop's ability to use rainfall that occurs in the fall for biomass accumulation without the added cost and delay involved in rebuilding the canopy. The value of the VPD-limited transpiration trait was also found to be higher in environments with more elevated VPD and lower rainfall. Examining the rainfall distribution and its effect on VPD-limited transpiration trait impacts showed that in years when water supply was low during the summer months, genotypes with *m*_2_ = −12.49 gained 20% more biomass than genotypes lacking a *vpd*_*BP*_. However, in years with higher summer rainfall these genotypes generate 10% less biomass, illustrating the trade-off associated with this trait (Supplemental Table 1).

Efforts to identify optimal trait ideotypes for specific crops and environments has been promoted as a way to speed up the rate of crop genetic improvement. For example, a maize root ideotype that is steep, deep and cheap has been identified and used to guide genotype selection and testing (Lynch, 2013). Consideration of the benefits and trade-offs of VPD-limited transpiration in energy sorghum in this study suggest that a beneficial ideotype would combine; (i) a high *m*_1_ and rapid leaf area development during the early portion of the season to maximize biomass accumulation under conditions of low VPD and high water supply and, (ii) induction of a VPD-limited transpiration trait (*m*_2_ < −200) later in the season when VPD is higher, possibly in response to water deficit. Prior research showed that traits that limit transpiration can be environmentally triggered by changes in VPD (Lobet et al., 2014; Vadez et al., 2014; McAdam and Brodribb, 2015), by altering the level/activity of root aquaporins (Choudhary and Sinclair, 2014a), and by foliar accumulation of the phytohormone abscisic acid (ABA) (Assmann et al., 2000; Bauer et al., 2013; McAdam and Brodribb, 2015; McAdam et al., 2015) that reduces stomatal aperture and plant transpiration (Tardieu and Davies, 1992; Parent et al., 2009; Boyer, 2010; Cutler et al., 2010). Stomatal closure can be further engineered via the ABA pathway, whereby application of agrochemicals on plants expressing genetically engineered ABA receptors drastically reduces transpiration on demand (Park et al., 2015). Genetic engineering approaches like these may provide a means to design plants with inducible and more negative *m*_2_ that enhance biomass yield in water limited environments. This type of genotype would be useful for environments that experience summer water deficit or terminal drought.

Determining target production environments for energy sorghum with VPD-limited transpiration will be critical for capturing the utility of the trait as well as in screening for energy sorghum hybrids that exhibit VPD-limited transpiration (Tardieu, 2012). Given the ideotype described above, selection for genotypes with high rates of LAI development and biomass accumulation during the first 75 DAS, combined with selection for genotypes that show delayed leaf senescence under water limiting conditions could be employed. Numerous QTL for stay-green have been identified in sorghum, and a subset of these loci may modulate VPD-traits that restrict transpiration at high VPD. High resolution yield mapping, and landscape models are being used to optimize crop productivity, yield stability, and return on investment by considering alternative distributions of available crops on perennial and annual cropland. For example, analysis of production regions in the US mid-west identified the potential utility of *Miscanthus*, a high biomass perennial C4 grass, for perennial cropland (Eranki et al., 2013). In a similar way, energy sorghum, with its high biomass yield, water use efficiency and resilience to water deficit could be usefully deployed in annual cropland landscapes, including sub-portions of fields that have shallow soils that are more often subject to water deficit. The APSIM energy sorghum crop model could be used in conjunction with landscape design modeling to optimize utilization of this new high biomass crop.

In conclusion, this study (i) demonstrated that modeling of energy sorghum in the APSIM framework is able to track field growth and biomass accumulation in different environments, (ii) extended the daily APSIM-sorghum model to incorporate hourly VPD-limited transpiration, (iii) found that VPD-limited transpiration can improve crop productivity in water-limiting environments by increasing TE and by maintenance of crop canopies to enable utilization of sporadic rainfall, and (iv) this model is made available for further evaluation of bioenergy sorghum production in other targeted environments and for other traits such as facultative CAM (Borland et al., 2009) that could further enhance water use efficiency and drought resilience.

## Author contributions

ST developed and integrated the VPD-limited transpiration model. ST and RM parameterized the sorghum bioenergy crop model. ST, RM, and JM designed the experiments, analyzed the data, and wrote the manuscript.

## Funding

This research was supported by the U.S. Department of Energy (DOE), Office of Science, Biological and Environmental Research (BER), award numbers DE-SC0012629 and DE-FC02-07ER64494 (Great Lakes Bioenergy Research Center), and by the U.S. DOE, Advanced Research Projects Agency-Energy (ARPA-E), award number DE-AR0000596. The views and opinions of authors expressed herein do not necessarily state or reflect those of the United States Government or any agency thereof.

### Conflict of interest statement

The authors declare that the research was conducted in the absence of any commercial or financial relationships that could be construed as a potential conflict of interest.
