# Supplementary material for: Bioenergy Sorghum Crop Model Predicts VPD-Limited Transpiration Traits Enhance Biomass Yield in Water-Limited Environments
Source: Front Plant Sci. 2017 Mar 21;8:335. doi: 10.3389/fpls.2017.00335 (PMC5359309; doi:10.3389/fpls.2017.00335)
Supplement: Supplementary file 1 [file DataSheet1.PDF]

Supplementary Material

Bioenergy sorghum crop model predicts VPD-limited transpiration traits enhance biomass yield in water-limited environments

Sandra K. Truong[1,2], Ryan F. McCormick[1,2], and John E. Mullet[1,2,*]

[1] Interdisciplinary Program in Genetics, Texas A&M University, College Station, Texas, 77843

[2] Department of Biochemistry and Biophysics, Texas A&M University, College Station, Texas, 77843

*** Correspondence:**
John E. Mullet
jmullet@tamu.edu

1 Supplementary Data

1.1 VPD-limited transpiration in simulated water-environments

The VPD-limited transpiration trait model was evaluated in simulated terminal-drought and well-watered environments for its duration in the vegetative stage in order to examine the trade-offs dependent on water in this relatively long developmental stage. Energy sorghum canopy closure occurs ~75 days after emergence and remains closed until harvest when the crop has sufficient water supply to maintain an LAI > 4 (~200 days). Therefore, modeling was initially used to observe the effects of the five m_2 values on biomass accumulation during the summer in College Station, Texas, at a developmental stage where all genotypes have closed canopies (LAI > 4). To aid examination of the impact of variation in m_2, the VPD-limited transpiration trait was induced after canopies had fully developed under fully irrigated conditions so that all genotypes modeled were equivalent until the point of trait induction. After the trait was induced, no additional water was provided to simulate a terminal-drought condition and to observe differences in how the genotypes utilized water in the soil profile in the absence of additional irrigation or rainfall (Supplemental Figure 3C-E). This analysis shows that the rate of biomass accumulation per day declines as m_2 decreases and the total biomass accumulated increases due to increased transpiration efficiency, resulting in an increase in water use efficiency (Supplemental Figure 3C, E). This trade-off occurs because the VPD-limited transpiration trait shifts plant water utilization and biomass accumulation to times of day with lower VPD where transpiration efficiency, $\frac{TE_c}{vpd(t)}$, is higher. The simulated genotype lacking a vpd_{BP} accumulated biomass the most rapidly and used 90% of the water available for biomass accumulation within ~13 days. In contrast, the genotype with the most restricted transpiration rate at VPD higher than the breakpoint accumulated biomass most slowly, using 90% of the available water in the soil profile in ~44 days. The restriction of water uptake and use to times of the day when transpiration efficiency was higher resulted in more biomass accumulated under water-limited conditions. A comparison of genotypes with the most extreme m_2 parameters evaluated ($m_2 = -12.49, 20.26$) illustrates this response. Plants where $m_2 = -12.49$ take ~30 days longer to utilize available water in the soil profile, accumulate an additional 1.235 kg m^{-2}, approximately 32% more than plants lacking a vpd_{BP}, $m_2 = 20.26$, which accumulate an additional 0.937 kg m^{-2} after irrigation stops (Supplemental Figure 3C). The additional gain associated with an $m_2 = -12.49$ during the simulated terminal-drought resulted in an overall biomass increase of 18% relative to the plants lacking a vpd_{BP}. Moreover, leaf senescence occurs in sorghum when water limiting conditions induce water stress, which physically reduces transpirational surface area (Blum, 2004). The model includes a water deficit induced leaf senescence response, therefore decreases in LAI occur most rapidly in in genotypes with large m_2 (Supplemental Figure 3D). In contrast to the terminal-drought condition, the VPD-limited transpiration trait has a negative impact on biomass accumulation under water sufficient conditions (Supplemental Figure 3F-H). To assess this potential loss of biomass, the impact of the VPD-limited transpiration trait was modeled by activating the response following canopy closure as described above in the simulated terminal-drought, except in in a well-watered condition. The results show that under well-watered conditions, genotypes that have the VPD-

limited transpiration trait accumulated less biomass, and this impact was greatest on genotypes with the most negative breakpoints (i.e. $m_2 = -12.49$) (Supplemental Figure 3F-H). These results indicate that utilization of the VPD-limited transpiration trait in breeding and deployment of genotypes that express the trait need to be optimized for target environments, and modeling represents a means to identify these optima for further testing.

Blum, A. (2004). Sorghum physiology. *Physiology and Biotechnology Integration for Plant Breeding, CRC Press, Boca Raton, FL, USA*, 141-224.

2 Supplementary Figures and Tables

2.1 Supplementary Figures

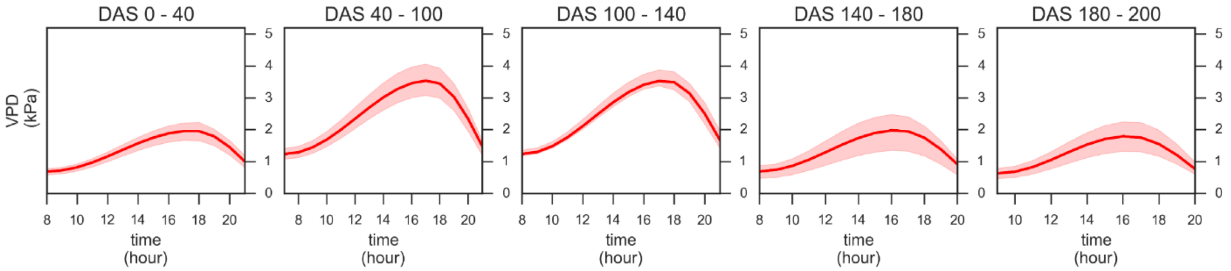

Supplemental Figure 1. Daily variation of VPD parameters for cropping conditions of College Station, TX, in 2009. The VPD mean for the DAS ranges is plotted in a solid red line and the more transparent range contains 75% of the data.

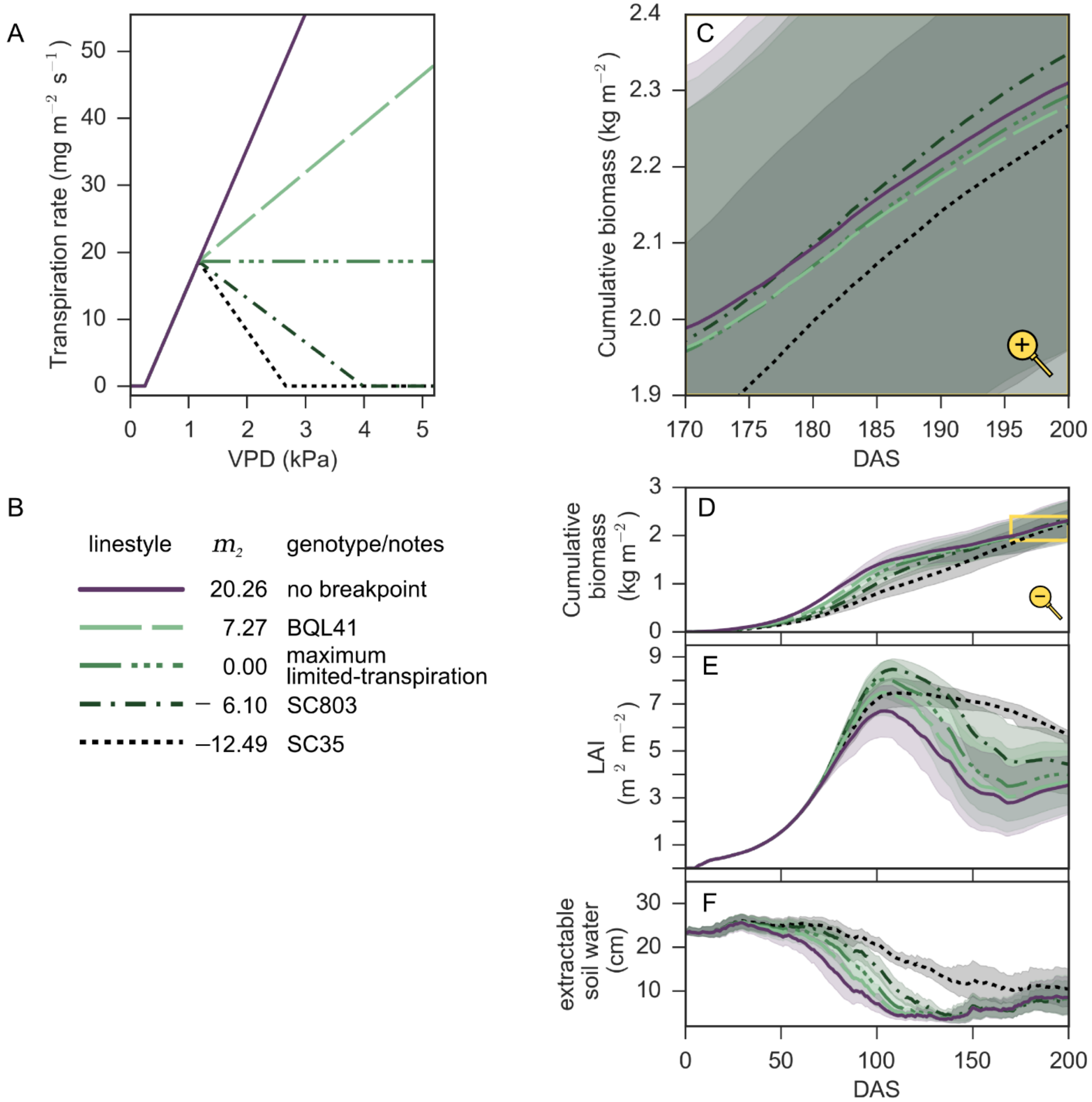

Supplemental Figure 2. Predictions of biomass accumulation by bioenergy sorghum with VPD-limited transpiration traits grown in rain fed cropping conditions of College Station, TX, 2000 – 2014 against days after sowing (DAS). (A, B) Diagram of the parameters of VPD-limited transpiration, with variation of transpiration rate after VPD breakpoint, vpd_{BP}, of 1.17 kPA (representative of the BQL41 genotype); $m_2 \in \{20.26, 7.27, 0, -6.1, -12.49\}$. (C-G) The simulations start with a fully saturated water profile at DAS 0. Light bands are 98% confidence interval bands from 50,000 bootstraps. (C) Zoomed in biomass accumulation for DAS 190 – 200 of panel D. Simulations of (D) biomass (E) LAI and (F) extractable soil water are plotted against DAS 0 – 200.

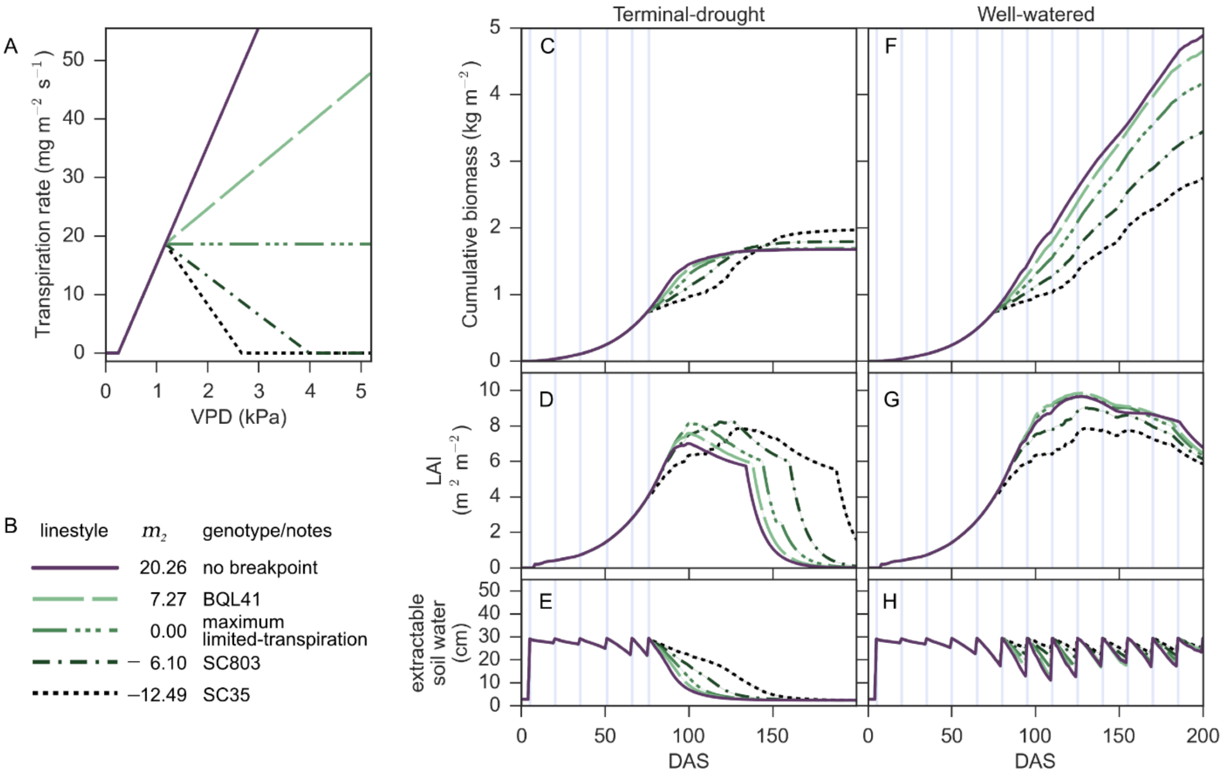

Supplemental Figure 3. Predictions of biomass accumulation by bioenergy sorghum with VPD-limited transpiration traits grown in simulated terminal drought and well-watered environments. (A) Diagram of the parameters of VPD-limited transpiration, with (B) variation of transpiration rate response to VPD after VPD breakpoint, vpd_{BP}, of 1.17 kPA (representative of the BQL41 genotype); $m_2 \in \{20.26, 7.27, 0, -6.1, -12.49\}$. Simulations of crop physiology where the VPD-limited transpiration trait is induced on day 75 (after sowing; day 0 after canopy closure, LAI > 4) with a fully saturated water profile. The divergence of the VPD-limited transpiration plants illustrates the rate and efficiency of water use of the different responses to VPD. (C) Biomass accumulated, (D) leaf area index (LAI), and (E) extractable soil water in simulated terminal-drought after canopy closure. (F) Biomass accumulated, (G) LAI, and (H) extractable soil water in simulated well-watered conditions.

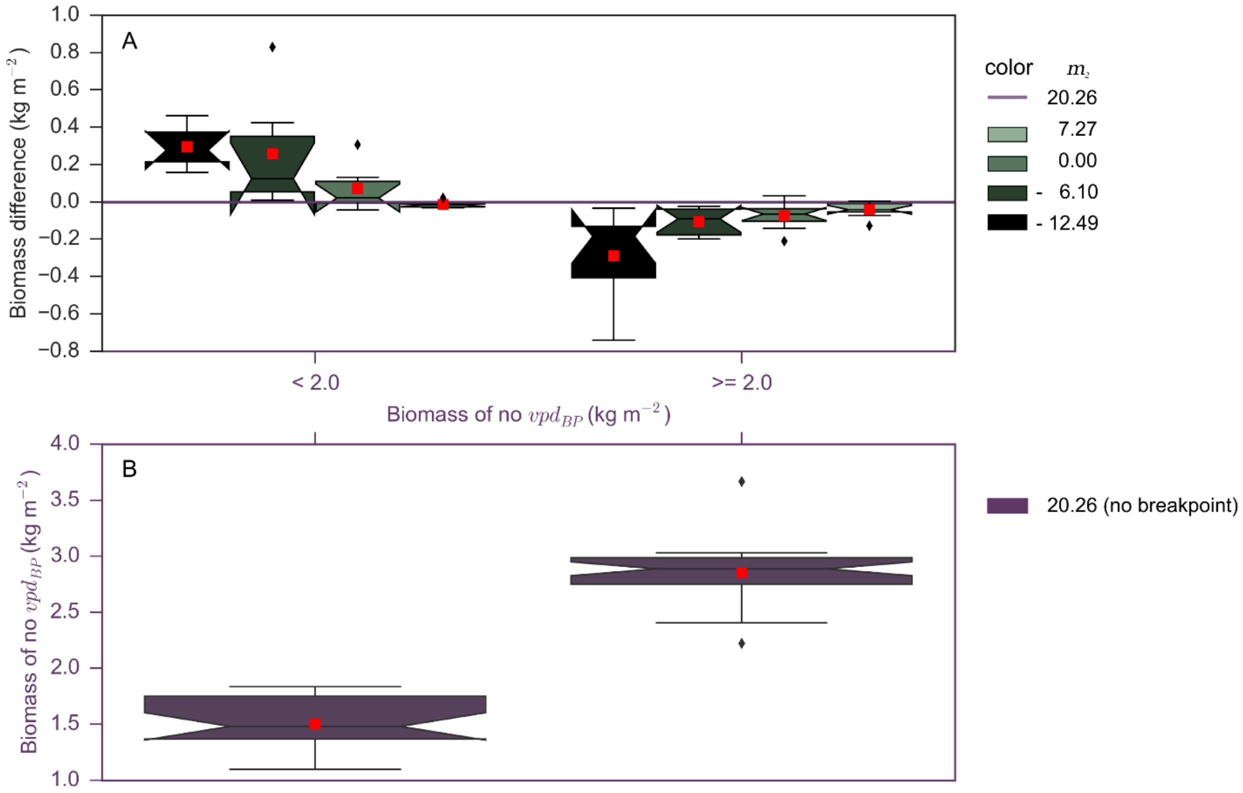

Supplemental Figure 4. Performance of VPD-limited transpiration in low and high yielding rain-fed cropping environments. (A) Boxplots show the biomass difference between genotypes with a VPD-limited transpiration trait and the genotype that lacks a vpd_{BP} ($m_2 = 20.26$). The notches represent the 95% confidence interval of the median, and the red boxes are the respective group means. These statistics are plotted against end biomass accumulated (DAS 200) by the genotype that lacks a vpd_{BP}, where the low and high yielding environments are defined as above and below 2.0 kg m^{-2}. (B) Boxplots of average biomass yields for plants that lack a vpd_{BP} in low and high yielding environments.

2.2 Supplementary Tables

Supplemental Table 1. Environmental data and end biomass of energy sorghum that vary in their m_2 simulations in the rain-fed cropping environments of College Station, TX. The table gives data for all years evaluated (2000 – 2014), as well as further separation to low-yielding (2000, 2005, 2009, 2011 – 2013) and high-yielding (2001 – 2004, 2006 – 2008, 2010, 2014) years that are determined by the drought condition in Figure 5. The VPD-limited transpiration parameters that are fixed here reflect that of BQL41 genotype ($m_1 = 20.26;\ vpd_{BP} = 1.17$).

Years	End biomass of no vpd_{BP}	Daily VPD	Yearly rain	m_2	Difference in end biomass: $m_2 -$ no vpd_{BP}	
(yield)	(kg m^{-2})	(kPa)	(cm)	(mg H_2O m^{-2} s^{-1} kPa^{-1})	(kg m^{-2})	(%)
2000 – 2014	2.31 ± 0.74	3.68 ± 0.41	56 ± 18	7.27	-0.03 ± 0.04	- 1.34
				0	-0.02 ± 0.12	- 0.74
				- 6.1	$+0.04 \pm 0.26$	1.66
				-12.49	-0.06 ± 0.35	- 2.42
2000, 2005, 2009, 2011 – 2013	1.50 ± 0.24	4.03 ± 0.34	43 ± 17	7.27	-0.01 ± 0.02	- 0.96
				0	$+0.07 \pm 0.12$	4.72
				- 6.1	$+0.26 \pm 0.29$	17.11
				-12.49	$+0.30 \pm 0.12$	19.64
2001 – 2004, 2006 – 2008, 2010, 2014	2.85 ± 0.39	3.44 ± 0.23	65 ± 13	7.27	-0.04 ± 0.04	- 1.47
				0	-0.08 ± 0.07	- 2.65
				- 6.1	-0.12 ± 0.07	- 3.77
				-12.49	-0.29 ± 0.24	- 10.17
